# Food insecurity and intimate partner violence among married women in Nepal

**DOI:** 10.7189/jogh.09.010412

**Published:** 2019-06

**Authors:** Nadia Diamond-Smith, Amy A Conroy, Alexander C Tsai, Manali Nekkanti, Sheri D Weiser

**Affiliations:** 1 University of California, San Francisco, California, USA; 2 Harvard University, Cambridge, Massachusetts, USA; 3 University of North Carolina, Chapel Hill, North Carolina, USA

## Abstract

**Background:**

Intimate Partner Violence (IPV) is an important public health concern globally, including in Nepal. Food insecurity (being without reliable access to a sufficient quantity of affordable, nutritious food) has been associated with IPV, but no known studies have explored this relationship in South Asia, or Nepal specifically. Women’s level of empowerment is an important factor to consider when understanding the relationship between food insecurity and IPV.

**Methods:**

Using data from the 2011 Nepal Demographic and Health Survey, we explore the relationship between different levels of food insecurity (none, mild, moderate, severe) and three types of IPV: physical, sexual and emotional. In a second set of models we adjust for indicators of women’s empowerment. We use multi-variable logistic regression to test for an association between these factors, adjusting for individual and household level demographic variables.

**Findings:**

About half of married women in our sample experience food insecurity and approximately 10% of women experienced each of the three different types of IPV in the past 12 months: emotional, sexual and physical. Food insecurity is significantly associated with increased odds of experiencing emotional (odds ratio OR = 1.75 95% confidence interval CI = 1.06-2.77 for severe food insecurity) or physical (OR = 2.48, 95% CI = 1.52-4.04 for severe food insecurity) IPV, but not sexual IPV, after adjusting for individual and household level demographic variables. After adjusting for empowerment related factors, this relationship still holds, although it is somewhat attenuated. Women’s level of household decision-making power is significantly associated with higher odds of emotional, sexual and physical IPV, and whether she lives with her in-laws is protective against emotional IPV.

**Conclusions:**

Among married women in Nepal, being food insecure is associated with higher odds of some types of IPV, specifically emotional and physical IPV. Accounting for women’s level of empowerment explains some of the relationship between food insecurity and IPV. It is essential that interventions to prevent IPV address household stressors such as food insecurity among married, Nepalese women, perhaps through cross-sectoral approaches. Such structural interventions are likely to reduce IPV for married women across South Asia who live in a similar levels of gender discrimination and food insecurity.

Intimate partner violence (IPV) is associated with a wide range of adverse mental and physical health outcomes, and is a major human rights concern worldwide. These adverse sequelae include poor physical health (eg, injuries), reproductive and sexual health (eg, unplanned pregnancy, abortion, maternal mortality, STIs/STDs, HIV) and psychological well-being (eg, suicidal thoughts and attempts, depression and anxiety) [1-5]. In Nepal, women experience high levels of IPV, with the most recent Nepal Demographic and Health Survey in 2011 (NDHS) finding that 28.31% of married women experienced some form of IPV in the last 12 months [6]. Among Nepalese women, sexual IPV has been associated with body aches, vaginal bleeding and thoughts of suicide [7]. IPV among pregnant women in Nepal has also been associated with lower rates of breastfeeding [8], potentially leading to the intergenerational transmission of health and social disadvantage.

Many community and household level factors are associated with IPV, including poverty and other factors that contribute to the disempowerment of women. Food insecurity is an important, yet understudied, risk factor for IPV in both developed and developing countries [1,9,10]. About half of all women in the NDHS 2011 reported some level of food insecurity, with 14% of all respondents reporting severe food insecurity [11]. However, research on the association between food insecurity and IPV is a relatively new area of investigation. Most studies on the association between food insecurity and IPV have utilized convenience samples (such as those receiving public assistance) rather than population-based samples which could allow for broader generalizability [10,12,13]. Moreover, the majority of studies have focused on understanding food insecurity and IPV within higher-income countries such as the US, Canada, and United Kingdom [14,15]. One study in Brazil found that food insecurity was associated with physical IPV [16]. Specifically in Asia, a multi-country study found that food insecurity was positively associated with the perpetration of both sexual and physical violence in two of the nine countries studied (Cambodia and Papua New Guinea) [17]. Notably, no studies have examined this association in Nepal, a country with alarmingly high rates of both food insecurity and IPV.

Drawing upon the broader literature, there are a number of different pathways that may explain how food insecurity could impact women’s experiences of sexual, physical, and emotional IPV. First, women who are food insecure may be more vulnerable to abuse and likely to stay in violent partnerships for the provision of food. In Nepal, women face greater obstacles to divorce than men for many reasons including beliefs that a divorced woman is “impure” [18]. However, there is evidence to suggest that women with higher education (and by extension, higher wealth and food security) may have more egalitarian relationships [19,20] that are protective against IPV. Second, women in violent relationships may be more likely to be food insecure if perpetrators of violence also control women’s access to food [15]. In Nepal, micronutrient deficiencies in women have been attributed to their lower status in the household. Foods containing higher nutrients are often preferentially allocated to adult males and children [21]. Third, food insecurity may be related to violence through the pathway of stress and poor mental health. For example, the stress caused by hunger and having insufficient resources for food could serve as a trigger for couple conflict and violence [22,23]. Research from other settings suggests that poor nutritional status caused by food insecurity [24] contributes to mental health conditions such as depression [25] which in turn may contribute to increased marital distress and violence [26,27].

A potential confounder in this association is women’s empowerment. A wide array of individual and household-level factors associated with women’s status and empowerment have been found to increase a woman’s likelihood of experiencing IPV in Nepal. These factors include young age at marriage, low educational status of the woman and her husband, low female autonomy (often measured by decision-making), low household economic status, husband’s alcohol consumption, male dominance and controlling behaviors towards women, number of children, being of lower caste/ethnic group, and living in the Terai region of the country [6,28]. In Nepal, food insecurity may be especially related to women’s status due to the common practice of women eating after men or separate from men [29]. Additionally, lower-status women (such as women married to younger compared to older brothers) in Nepal have more limited access to household food and nutritional resources [11,30]. Given that both IPV and food access have been associated with lower household status for women, it is possible that women’s status in the household, food insecurity, and IPV may be associated in this setting. Thus, this paper contributes to furthering the understanding of this relationship using nationally-representative, population-based data from Nepal to confirm these findings. Additionally, we aim to better understand additional factors may be involved in the association between food insecurity and IPV.

In this study, the primary objective is to investigate the association between food insecurity and three measures of IPV (physical, sexual, and emotional) among married women in Nepal. As part of this goal, we will also test whether the association between food insecurity and IPV remains even after controlling for women’s status and empowerment in the household. We hypothesize that women who live in households that are food insecure will be more likely to report IPV and that the association will be persist after controlling for measures of women’s status and empowerment. We accomplish these objectives using data from the 2011 Nepal Demographic and Health Survey (NDHS 2011).

## METHODS

### Study procedures

We used data from the woman’s and household questionnaires of the Nepal Demographic and Health Survey of 2011 (NDHS 2011). The NDHS sampling and recruitment is described in detail elsewhere, but briefly, it uses a stratified, two-staged cluster design to collect data on women of reproductive age (15-49 years) and a subset of their husbands [31]. Because only a random subset of married women were asked the questions about experience of violence, we restricted the analysis to married women who were administered the IPV module (N = 3373).

### Measures

#### Sexual, physical, and emotional IPV

Three different binary measures of intimate partner violence were created from a set of questions asked to a subset of female respondents in the NDHS about their experiences of different types of IPV in the last 12 months: sexual, physical, and emotional. Sexual IPV was ascertained with two questions: “Have you ever been physically forced into unwanted sex by your husband /partner?” and “Have you ever been forced into other unwanted sexual acts by your husband/partner?”. Physical IPV was ascertained with seven questions such as “Have you ever been kicked or dragged by your husband/partner?” and “Have you ever been pushed, shook or had something thrown by your husband/partner?”. Emotional IPV was ascertained with three questions such as “Have you ever been humiliated by your husband/partner?” and “Have you ever been threatened with harm by your husband /partner?” Three binary variables were created for the three forms of IPV (yes/no). Women who responded “sometimes” or “often” to any of the questions were coded as having experienced that particular form of IPV.

#### Food insecurity

Household food insecurity was measured using the Household Food Insecurity Access Scale, developed by the USAID’s Food and Nutrition Technical Assistance (FANTA) project [9,32]. Questions asked about insufficient quantity and quality of foods, and anxiety and uncertainty about food supply. The scale was modified slightly for the Nepalese context and demonstrated good reliability in this setting. Food insecurity questions were ascertained at the household-level and were generally answered by the head of the household (generally a male in this setting). A summary score was constructed based on the 7 questions included. Then, as is standard practice, food insecurity scores were categorized into four levels of increasing food insecurity: food secure (1), mild food insecurity (2), moderate food insecurity (3) and severe food insecurity (4).

#### Confounding variables

We adjusted for variables related to women’s status and empowerment based on previous literature suggesting that these variables may be associated with IPV and/or food insecurity in Nepal. These variables included fertility (whether the respondent had a son; yes/no), total number of living children (continuous variable), and pregnancy status (yes/no) [6,33]. Women who were married at young ages have been found to have lower status, thus we included a continuous variable for age at marriage [34]. As an indicator for autonomy, we included a household decision-making score (continuous variable) comprised of five questions related to a woman’s ability to make decisions about domains such as visiting family friends and how to spend their own earnings [34]. Finally, we included a variable indicating whether the respondent lives in a joint household, defined as living with at least one other sister-in-law and a mother-in-law (yes/no) [11].

In addition, we included a set of individual and household-level covariates based on previous literature suggesting that these variables may confound the relationship between food insecurity and IPV [6,28]. These included women’s age (in 5-year age groups), caste/ethnicity (Brahmin/Chhetri, Newar, Dalit, Janajati, or other), whether the female respondent was the household head (yes/no), if the respondent lived with her husband (yes/no), educational attainment of both the woman respondent and her husband (none, primary, secondary, and higher than secondary), occupation (professional occupation vs other), geographic region (mountains, hills or Terai), development region (eastern, central, western, mid-western, far-western), urban/rural status, and wealth quintiles [35,36]. In addition, we included a binary variable about whether the husband consumes any alcohol currently (yes vs no), as previous literature has found this to be associated with IPV [6].

### Analysis plan

In the first set of models, we examined the relationship between food insecurity and the three IPV variables (sexual, physical, and emotional IPV). We fit a series of unadjusted logistic regression models and then re-fit the models adjusting for individual and household-level covariates. Next we added in the variables for women’s status and empowerment, resulting in the final, multivariable models. Data were analyzed using STATA 15 (StataCorp, College Station, TX) and included survey weights for all analyses. Differences were considered statistically significant at *P* < 0.05.

## RESULTS

A total of 3373 women were included in our analysis, as they had data about both IPV and food insecurity. Approximately 9% of women reported experiencing sexual IPV, 11.9% reported experiencing physical IPV, and 11.4% reported experiencing emotional IPV in the last 12 months (**Table 1**). Almost half (49.4%) of women lived in food secure households, with 13.6% living in mildly food insecure, 21.4% in moderately food insecure, and 15.6% in severely food insecure households.

**Table 1:** Percent and number of women and food insecurity in the last 12 months (N = 3373)

	Percent	N (unadjusted for survey weights)
**Age (years):**		
15-19	6.67	225
20-24	17.97	606
25-29	22.74	767
30-34	19.03	642
35-39	14.91	503
40-44	10.55	356
45-49	8.12	274
**Caste/ethnicity:**		
Brahmin/Chhetri	42.22	1424
Newar	3.88	131
Dalit	15.21	513
Janajati	30.74	1037
Other	7.95	268
**Respondent is household head**	21.61	729
**Lives with partner**	67.98	2293
**Education level:**		
No education	46.93	1583
Primary	18.89	637
Secondary	28.11	948
Higher	6.08	205
**Professional occupation (compared to non-professional or no occupation)**	3.79	128
**Husband drinks alcohol**	53.90	1818
**Region:**		
Mountain	16.84	568
Hill	39.61	1336
Terai	43.55	1469
Urban (compared to rural)	26.86	906
**Development region:**		
Eastern	23.39	789
Central	23.48	792
Western	17.46	589
Mid-western	19.27	650
Far-western	16.39	553
**Mean decision score (range 0-5)**	1.16	
**Has at least 1 son**	75.63	2551
**Mean number of children (range 0-8)**	2.38	
**Currently pregnant**	6.48	613
**Mean age at marriage (range 9.6-35.75)**	17.23	
**Lives in a joint households**	26.15	2474
**Experienced IPV in last 12 months:**		
Emotional IPV, past 12months	11.4	383
Sexual IPV, past 12 months	9.1	306
Physical IPV, past 12 months	11.9	400
**HFIAS category:**		
Food Secure	49.42	1667
Mild FI	13.64	460
Moderate FI	21.35	720
Severe FI	15.59	526

HFIAS – Household Food Insecurity Access Scale, FI – food insecurity, IPV – intimate partner violence

### Association between food insecurity and IPV

In the unadjusted analyses, living in a household with moderate or severe food insecurity was significantly associated with increased odds of sexual IPV (**Table 2**). Living in a household with mild, moderate, or severe food insecurity was significantly associated with increased odds of physical and emotional IPV. After adjusting for individual and household-level factors, living in a household with mild (OR = 2.10, 95% CI = 1.34-3.31), moderate (OR = 1.50, 95% CI = 0.85-2.35), or severe (OR = 1.93, 95% CI = 1.19-3.12) food insecurity was associated with increased odds of emotional IPV. Living in a household with mild (OR = 2.82, 95% CI = 1.76-4.52), moderate (OR = 1.68, 95% CI 1.09-2.60) or severe (OR = 2.79, 95% CI = 1.67-4.65) food insecurity was associated with an increased odds of physical IPV. Food insecurity was not significantly associated with sexual IPV in adjusted models.

**Table 2:** Associations (odds ratio, 95% confidence interval) between food insecurity and past year IPV, by type and severity of IPV

IPV Outcome	Food insecurity	Univariate N = 3373	Multivariate* N = 3357
Emotional IPV, past 12 months	Mild food insecurity	2.154 (1.371-3.386)§	2.101 (1.335-3.306)§
	Moderate food insecurity	1.736 (1.176-2.563)§	1.501 (0.991-2.274)†
	Severe food insecurity	2.578 (1.808-3.676)§	1.929 (1.192-3.123)§
Sexual IPV in the last 12 months	Mild food insecurity	1.509 (0.927-2.455)†	1.412 (0.848-2.352)
	Moderate food insecurity	1.772 (1.106-2.840)‡	1.548 (0.927-2.585)†
	Severe food insecurity	1.735 (1.190-2.531)§	1.351 (0.829-2.200)
Physical IPV in the last 12 months	Mild food insecurity	2.703 (1.727-4.232)§	2.818 (1.759-4.515)§
	Moderate food insecurity	1.944 (1.248-3.026)§	1.680 (1.086-2.597)‡
	Severe food insecurity	3.701 (2.486-5.509)§	2.786 (1.669-4.653)§

IPV – intimate partner violence

*Controlling for woman’s age, woman and husband education, ethnicity/caste, geographic region of the country, urban/rural status, development region, woman is the household head, living with partner, occupation of woman and household wealth index.

†*P* < 0.1.

‡*P* < 0.05.

§*P* < 0.01.

### Adjusting for women’s status and empowerment

After adjusting for women’s status variables as potential confounders (in addition to the covariates above), a non-significant association remained between any level of food insecurity and sexual IPV (**Table 3**). After adjusting for potential confounders related to women’s status, mild (OR = 2.14, 95% CI = 1.30-3.54) and severe (OR = 1.72, 95% CI = 1.06-2.77) food insecurity remained associated with increased odds of a women experiencing emotional IPV. Physical IPV remained associated with mild (OR = 3.01, 95% CI = 1.80-5.06) and severe (OR = 2.48, 95% CI = 1.52-4.04) food insecurity, after adjusting for women’s status variables. Having a higher score on the women’s decision-making variable was also associated with increased odds of emotional, sexual, and physical IPV. Living in a co-resident household was protective against emotional IPV, but not sexual or physical IPV. None of the other women’s status variables were associated with any form of IPV.

**Table 3:** Association (odds ratio, 95% confidence interval) between food insecurity and IPV, controlling for women’s status variables (N = 3003)*

	Emotional IPV in the last 12 months	Sexual IPV in the last 12 months	Physical IPV in the last 12 months
Mild food insecurity	2.141 (1.295-3.541)§	1.570 (0.894-2.756)	3.013 (1.796-5.056)§
Moderate food insecurity	1.263, (0.802-1.988)	1.534, (0.882-2.667)	1.503 (0.942-2.399)†
Severe food insecurity	1.715 (1.063-2.766)‡	1.421 (0.797-2.534)	2.476, (1.518-4.038)§
Household decision-making score	1.205 (1.082-1.343)§	1.115 (0.985-1.261)†	1.200 (1.073-1.342)§
Has at least 1 son	0.892 (0.584-1.362)	1.057 (0.686-1.627)	1.112, (0.699-1.770)
Total number of children	1.000 (0.860-1.162)	1.040 (0.879-1.232)	1.078, (0.914-1.273)
Is currently pregnant	1.179 (0.705-1.974)	1.269(0.608-2.649)	1.611 (0.812-3.196)
Age at marriage	0.972 (0.904-1.045)	0.974 (0.907-1.046)	0.989 (0.926-1.057)
Lives in a co-resident household	0.560 (0.332-0.943)‡	0.582 (0.292-1.161)	0.933 (0.556-1.563)

*Controlling for woman’s age, woman and husband education, ethnicity/caste, geographic region of the country, urban/rural status, development region, woman is the household head, living with partner, occupation of woman, husband’s alcohol consumption, and household wealth index.

†*P* < 0.1.

‡*P* < 0.05.

§*P* < 0.01.

## DISCUSSION

We found that approximately 50% of married women in Nepal reported some level of food insecurity, while approximately 10% of women reported emotional, sexual and/or physical IPV in the last 12 months. Women who experienced food insecurity were more likely to experience emotional and physical IPV, even after adjusting for variables capturing different aspects of women’s status and empowerment. The association did not hold for sexual IPV in the multivariable models – suggesting that the associations between food insecurity and IPV may differ by type of IPV. We also found that mild food insecurity is significantly associated with emotional and physical IPV, but the relationship is not significant for moderate food insecurity. These findings suggest that when women live in households that are somewhat stressed by food insecurity they are most prone to experience IPV. The directionality and magnitude are similar for moderate food insecurity, the relationship just not significant. Perhaps in very food insecure households women are less likely to report IPV since they are experiencing more stress broadly.

It is possible that different forms of IPV affect women’s risk for IPV through different pathways. Women’s engagement in transactional sex in exchange for food may explain the association between food insecurity and sexual IPV, as shown in Uganda [37]. In this married sample of Nepalese women, cultural norms prohibiting women’s marital infidelity may mean there are lower rates of transactional sex with extra-marital partners. It is also possible that stressful situations around food insecurity may be more likely to trigger episodes of emotional and physical IPV than sexual IPV. Other researchers have distinguished between sexual and non-sexual violence which they argue should be conceptualized and interpreted as distinct events; for example, some sexual perpetrators of a certain typology may never perpetrate physical violence against their partners [38].

We next explored whether the association between food insecurity and IPV held after adjusting for women’s status and level of empowerment in the household. The association between severe food insecurity and emotional IPV was somewhat attenuated by adjusting for these factors. This suggests that women’s status in the household may explain part of the association between severe food insecurity and emotional IPV. Adjusting for women’s status variables strengthened the association between physical IPV and mild food insecurity. This suggests that even after accounting for women’s status in the household, there is an association between mild food insecurity and physical IPV.

As demonstrated elsewhere [39,40], women who reported that their husbands drank alcohol were more likely to report sexual, physical, and emotional IPV. In particular, there was a strong association between a husband’s drinking and physical IPV. Interestingly, women having a higher score on the decision-making scale, indicating that they had more decision-making power in the household, were more likely to report emotional, sexual, and physical IPV. Women received a higher score if they reported that they made certain decisions on their own, and it is possible this type of autonomy leads to more tensions in the household. If we had measured joint decision-making, which could be a better marker of women’s status, we may have found a negative association with IPV. Future research should explore this in more detail. Finally, living in a joint household, with other sisters-in-laws and in-laws, was protective for emotional IPV. Past research in Egypt found that co-residing with in-laws was associated with higher relationship quality between the wife and husband, and thus lower emotional IPV could be reflective of higher relationship quality in this setting as well. It is also possible that couples are less comfortable arguing about sensitive issues (such as infidelity) with relatives nearby for fear of gossip [41], thus leading to less emotional IPV.

This study has several limitations. Questions on food insecurity and experiences of IPV were both asked about the previous 12-month period. Thus, we do not know the directionality of the relationship. Both experiences may have been acute or may have occurred repeatedly in that time span – hence the interpretation is complex. The time bounds on the questions provide some narrowing of the window (eg, as compared to measures that assess lifetime history of IPV) and make us more confident that the two experiences occurred close to each other in time, and are potentially overlapping. More research should explore the direction of this relationship, preferably using longitudinal data such as ecological momentary assessment studies, which could potentially assess the temporal relationship between food insecurity and violence. Additionally, IPV is often underreported, which could affect the magnitude of these relationships. It is possible that sexual IPV may be normalized for married women in this context [42], and thus women may have underreported acts of sexual violence. Underreporting of sexual violence in household surveys and to the police has been documented as a challenge in general [43,44], including among studies of married women in Nepal [7]. Finally, we were only able to adjust for a select set of measures on women’s status in the household and it is possible that other factors such as physical mobility may be important for understanding women’s status or empowerment.

Despite these limitations, this study has many strengths. Few population-based data sources contain information about food insecurity or IPV, much less both. Nepal DHS is the only DHS survey that contains both of these indicators. Therefore our ability to explore these relationships was a unique opportunity and makes a valuable contribution to the literature. Furthermore, we were able to separately examine multiple types of IPV (as opposed to a combined measure of sexual, physical, and emotional IPV), which may be related to food insecurity through varied pathways. Future studies using longitudinal data would be more appropriate to identify these pathways and understand differences by IPV type. A final strength is that we were also able to control for variables related to women’s status and empowerment, which are also not routinely collected in many data sets, and are important, especially in the South Asian context.

## CONCLUSION

Household stressors, such as food insecurity, are associated with multiple forms of IPV among married women in Nepal. While women’s status in the household explains some of this relationship, food insecurity remained strongly associated with emotional and physical IPV after controlling for these variables. These findings are likely relevant for women living throughout South Asia and other regions with low women’s status. Given the complex and multilevel nature of the factors explored in this analysis (food insecurity, violence and women’s empowerment), structural or inter-sectoral interventions may be the best approach. There are a few examples of such approaches related to these topics, and a recent systematic review of structural interventions mostly involving women’s economic and social empowerment, to reduce IPV found that most had a positive impact on the primary outcomes of reducing IPV [45]. We were not able to identify previous interventions specifically addressing food insecurity and women’s status as a means of reducing IPV. However, one past intervention aiming to reduce IPV and HIV through structural programs with microfinance was not found to reduce food insecurity, although it did reduce IPV [46]. Interventions aiming to reduce IPV should address household food insecurity concerns, as these may be key factors placing women at risk for IPV.

## References

[1] Ellsberg M, Jansen HA, Heise L, Watts CH, Garcia-Moreno C (2008). Intimate partner violence and women’s physical and mental health in the WHO multi-country study on women’s health and domestic violence: an observational study.. Lancet.

[2] Krug EG, Dahlberg LL, Mercy JA, Zwi AB, Lozano L. World report on violence and health. 2002. Available: http://apps.who.int/iris/bitstream/10665/42495/1/9241545615_eng.pdf. Accessed: 8 September 2017.

[3] Tsai AC, Wolfe WR, Kumbakumba E, Kawuma A, Hunt PW, Martin JN (2016). Prospective study of the mental health consequences of sexual violence among women living with HIV in rural Uganda.. J Interpers Violence.

[4] Tsai AC, Tomlinson M, Comulada WS, Rotheram-Borus MJ (2016). Intimate Partner Violence and depression symptom severity among South African women during pregnancy and postpartum: Population-based prospective cohort study.. PLoS Med.

[5] Tsai AC, Weiser SD, Dilworth SE, Shumway M, Riley ED (2015). Violent victimization, mental health, and service utilization outcomes in a cohort of homeless and unstably housed women living with or at risk of becoming infected with HIV.. Am J Epidemiol.

[6] Atteraya MS, Gnawali S, Song IH (2015). Factors associated with intimate partner violence against married women in Nepal.. J Interpers Violence.

[7] Puri M, Tamang J, Shah I (2011). Suffering in silence: consequences of sexual violence within marriage among young women in Nepal.. BMC Public Health.

[8] Roginiel A. The Impact Of Intimate Partner Violence On Breastfeeding: A Demographic And Health Surveys Analysis Of India, Nepal And Timor-Leste. Public Health Theses. 2013 Jan 1; Available: http://elischolar.library.yale.edu/ysphtdl/1247. Accessed: 12 November 12 2018.

[9] Tsai AC, Leiter K, Heisler M, Iacopino V, Wolfe W, Shannon K (2011). Prevalence and correlates of forced sex perpetration and victimization in Botswana and Swaziland.. Am J Public Health.

[10] Chilton MM, Rabinowich JR, Woolf NH (2014). Very low food security in the USA is linked with exposure to violence.. Public Health Nutr.

[11] Diamond-Smith N, Raj A, Prata N, Weiser SD (2017). Associations of women’s position in the household and food insecurity with family planning use in Nepal.. PLoS One.

[12] Tolman RM, Rosen D (2001). Domestic violence in the lives of women receiving welfare: Mental health, substance dependence, and economic well-being.. Violence Women..

[13] Wehler C, Weinreb LF, Huntington N, Scott R, Hosmer D, Fletcher K (2004). Risk and protective factors for adult and child hunger among low-income housed and homeless female-headed families.. Am J Public Health.

[14] Melchior M, Caspi A, Howard LM, Ambler AP, Bolton H, Mountain N (2009). Mental health context of food insecurity: a representative cohort of families with young children.. Pediatrics.

[15] Power EM (2006). Economic abuse and intra-household inequities in food security.. Can J Public Health..

[16] Ribeiro-Silva R de C, Fiaccone RL, Barreto ML, Santana MLP, dos Santos SMC, da Conceiçăo-Machado MEP (2016). The association between intimate partner domestic violence and the food security status of poor families in Brazil.. Public Health Nutr.

[17] Fulu E, Jewkes R, Roselli T, Garcia-Moreno C (2013). Prevalence of and factors associated with male perpetration of intimate partner violence: findings from the UN Multi-country Cross-sectional Study on Men and Violence in Asia and the Pacific.. Lancet Glob Health.

[18] Niraula BB, Morgan SP (1996). Marriage Formation, Post-Marital Contact with Natal Kin and Autonomy of Women: Evidence from Two Nepali Settings.. Popul Stud..

[19] Jennings EA (2016). Predictors of marital dissolution during a period of rapid social change: Evidence from South Asia.. Demography.

[20] Stash S, Hannum E (2001). Who goes to school? Educational stratification by gender, caste, and ethnicity in Nepal.. Comp Educ Rev.

[21] Gittelsohn J, Thapa M, Landman LT (1997). Cultural factors, caloric intake and micronutrient sufficiency in rural Nepali households.. Soc Sci Med.

[22] Benson ML, Fox GL. Economic Distress, Community Context and Intimate Violence: An Application and Extension of Social Disorganization Theory. Office of Justice Programs, US Department of Justice.

[23] Ingoldsby BB, Smith SR, Miller JE. Exploring family theories. Roxbury Pub.; 2004.

[24] Olson CM (1999). Nutrition and health outcomes associated with food insecurity and hunger.. J Nutr.

[25] Bodnar LM, Wisner KL (2005). Nutrition and depression: implications for improving mental health among childbearing-aged women.. Biol Psychiatry.

[26] Chambliss LR (1997). Domestic violence: a public health crisis.. Clin Obstet Gynecol.

[27] Nunes MA, Ferri CP, Manzolli P, Soares RM, Drehmer M, Buss C (2010). Nutrition, mental health and violence: from pregnancy to postpartum Cohort of women attending primary care units in Southern Brazil-ECCAGE study.. BMC Psychiatry.

[28] Oshiro A, Poudyal AK, Poudel KC, Jimba M, Hokama T (2011). Intimate partner violence among general and urban poor populations in Kathmandu, Nepal.. J Interpers Violence.

[29] Gittelsohn J (1991). Opening the box: Intrahousehold food allocation in rural Nepal.. Soc Sci Med.

[30] Furuta M, Salway S (2006). Women’s position within the household as a determinant of maternal health care use in Nepal.. Int Fam Plan Perspect.

[31] Tsai AC, Weiser SD (2014). Population-based study of food insecurity and HIV transmission risk behaviors and symptoms of sexually transmitted infections among linked couples in Nepal.. AIDS Behav.

[32] Ministry of Health and Population [Nepal], New ERA, ICF International Inc. Nepal Demographic and Health Survey 2011. Kathmandu, Nepal: Ministry of Health and Population, New ERA, and ICF International, Calverton, Maryland; 2012.

[33] Deuba K, Mainali A, Alvesson HM, Karki DK (2015). Experience of intimate partner violence among young pregnant women in urban slums of Kathmandu Valley, Nepal: a qualitative study.. BMC Womens Health.

[34] Lamichhane P, Puri M, Tamang J, Dulal B (2011). Women’s status and violence against young married women in rural Nepal.. BMC Womens Health.

[35] Filmer D, Pritchett LH (2001). Estimating wealth effects without expenditure data–or tears: an application to educational enrollments in states of India.. Demography.

[36] Rutstein S, Johnson K. The DHS Wealth Index: Approaches for Rural and Urban Areas. Calverton, MD: ORC Macro; 2008.

[37] Miller CL, Bangsberg DR, Tuller DM, Senkungu J, Kawuma A, Frongillo EA (2011). Food insecurity and sexual risk in an HIV endemic community in Uganda.. AIDS Behav.

[38] Monson CM, Langhinrichsen-Rohling J (2002). Sexual and nonsexual dating violence perpetration: testing an integrated perpetrator typology.. Violence Vict.

[39] de Bruijn DM, de Graaf IM (2016). The role of substance use in same-day intimate partner violence: A review of the literature.. Aggress Violent Behav.

[40] World Health Organization. Preventing Violence by Reducing the Availability and Harmful Use of Alcohol. 2009. Available: http://www.who.int/violence_injury_prevention/violence/alcohol.pdf. Accessed: 25 September 2017.

[41] Conroy AA (2014). ‘It means there is doubt in the house’: perceptions and experiences of HIV testing in rural Malawi.. Cult Health Sex.

[42] Wilson-Williams L, Stephenson R, Juvekar S, Andes K (2008). Domestic violence and contraceptive use in a rural Indian village.. Violence Against Women..

[43] Ellsberg M, Heise L, Peńa R, Agurto S, Winkvist A (2001). Researching domestic violence against women: Methodological and ethical considerations.. Stud Fam Plann.

[44] Jewkes R, Abrahams N (2002). The epidemiology of rape and sexual coercion in South Africa: an overview.. Soc Sci Med.

[45] Bourey C, Williams W, Bernstein EE, Stephenson R (2015). Systematic review of structural interventions for intimate partner violence in low- and middle-income countries: organizing evidence for prevention.. BMC Public Health.

[46] Pronyk PM, Hargreaves JR, Kim JC, Morison LA, Phetla G, Watts C (2006). Effect of a structural intervention for the prevention of intimate-partner violence and HIV in rural South Africa: a cluster randomised trial.. Lancet.

